# Genomic Perspective on Heterogeneity of Organs and Body Aging

**DOI:** 10.1111/acel.70353

**Published:** 2026-01-02

**Authors:** Shabnam Salimi, Daniel Raftery, Luigi Ferrucci

**Affiliations:** ^1^ Translational Gerontology Branch, Biomedical Research Center National Institute on Aging, National Institutes of Health Baltimore Maryland USA; ^2^ Department of Anesthesiology and Pain Medicine University of Washington Seattle Washington USA; ^3^ Northwest Metabolomic Research Center University of Washington Seattle Washington USA

**Keywords:** epigenetics, genomics, Health Octo Tool, heterogeneity of aging, organ aging

## Abstract

Aging is a heterogeneous process, with organ systems and individuals experiencing variable rates of decline that are not fully reflected by chronological age. This variability contributes to the complexity of system morbidity, which poses increasing challenges for clinical care and biomedical research. In this review, we discuss the heterogeneity of organ and whole‐body aging and perspectives on genomics as possible mechanisms that relate to such heterogeneity. We discuss how static genomics, including nuclear genetic variants, and dynamic genetics, such as somatic mutations, epigenetic drifts, and mitochondrial DNA changes might explain the variable rate of aging across organ systems and the whole body. We discuss that the use of metrics that capture heterogeneity in organ and body aging is critical to identify genomic biomarkers of aging, clarifying mechanisms of adaptation versus decline.

## Graphical abstract

Organ‐specific and whole‐body intrinsic aging metrics reveal heterogeneous organ aging, which potentially can arise from the interplay between static germline variation, somatic mutations, epigenetic drift, transposable elements, and mitochondrial DNA changes, highlighting genomic pathways for precision, organ‐targeted aging interventions.

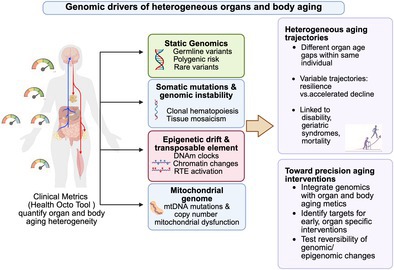

Recent research has demonstrated heterogeneity in organ and body aging by reframing aging as a multidimensional and system‐specific process and applying various tools to capture its heterogeneity (Goeminne et al. 2025; Oh et al. 2023; Salimi et al. 2025; Tian et al. 2023). Organ systems exhibit distinct biological aging rates, shaped by both intrinsic factors (e.g., genetics) and extrinsic exposures (e.g., environmental insults, lifestyle). Several independent yet converging approaches, ranging from proteomic aging clocks (Goeminne et al. 2025; Oh et al. 2023) to clinical metrics like age gap index (Tian et al. 2023), Bodily System‐Specific Clocks (BSC), Bodily System‐Specific Age (BSA), Body Clock, and Body Age (Salimi et al. 2025), consistently reveal substantial heterogeneity in the pace and features of aging at the organ and individual level. Moreover, integrating genomic data into clinical‐based models could shed light on the genetic underpinnings of aging and identify targets for personalized interventions.

In this review, we explore the contributions of static genetic variation (germline variants), and dynamic genomic alterations such as somatic mutations, epigenetic changes, and mitochondrial DNA (mtDNA) mutations and copy number alteration to the heterogeneity of organ aging. Such an approach has been shown in chronic disease onset and progression (Burns 2017; De Cecco et al. 2013; Lodato et al. 2018; Martincorena 2019) and the potential interplay between these mechanisms and the heterogeneity of intrinsic organ and body aging and their rate of aging (Kennedy et al. 2014; Lopez‐Otin et al. 2023). We briefly discuss methods used to show organ aging variability. We then explore potential genomic mechanisms that might underlie such variability and discuss how understanding this heterogeneity is critical to advancing personalized aging trajectories and targeted interventions.

## 1 Proteomic‐Based and Clinical‐Based Organ‐Aging Heterogeneity

**FIGURE 1:**
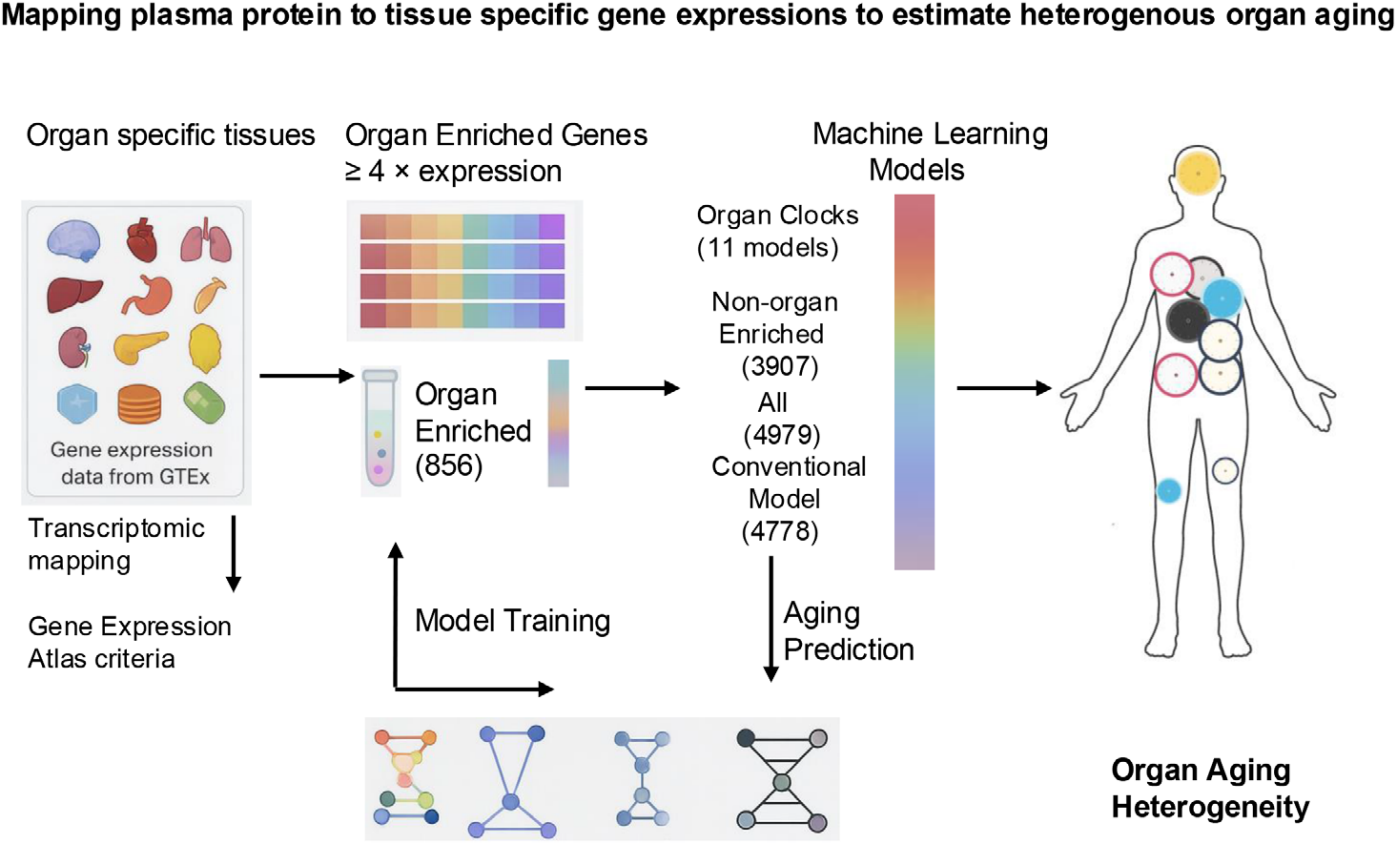
Framework for organ‐specific proteomic aging clocks. The graph illustrates the organ specific gene expressions used to map to plasma proteomics.

Oh et al. suggested that organ‐specific plasma proteins could serve as minimally invasive biomarkers to monitor aging across distinct 11 organs including adipose tissue, arteries, brain, heart, immune tissue, intestine, kidney, liver, lung, muscle, and pancreas (Oh et al. 2023). The investigators systematically mapped plasma proteins to tissue‐specific gene expression profiles using data from the Genotype‐Tissue Expression (GTEx) project (Figure 1), identifying tissue specificity based on a more than fourfold increase in gene expression associated with each protein (Table 1).

**TABLE 1:** Studies of proteomic‐ and clinical‐based organ aging heterogeneity.

Study	Study design sample size, data	Model	Predicted clinical outcomes	Conclusion
Oh et al. (2023)	Cross‐sectional (*n* = 5676), Training set (Knight Alzheimer's Disease Research Center *n* = 1398), LonGenity cohort: longitudinal (*n* = 812)	Trained chronological age on organ‐specific, organ‐non‐specific (organismal age), and all specific and nonspecific proteomics (conventional age)	9 diseases, 43 clinical biochemistry and cell count markers, mortality	Heterogeneity of organ aging across 11 organ systems, accelerated organ aging is associated with higher mortality risk, and organ‐specific diseases, individuals with the same conventional age gap had diverse organ aging profiles, low‐to‐moderate correlation between the age gaps of different organs. Age gaps of organs were associated with both organ‐specific and non‐organ specific diseases.
Goeminne et al. (2025)	Cross‐sectional UK Biobank (*n* = 44,952) Longitudinal (*n* = 985) Replicating in MESA (*n* = 921)	Trained chronological age and mortality on proteomics (conventional metrics) Trained chronological age and mortality on organ‐specific proteomics	ICD‐10‐based 10 diseases, mortality, grip strength	8 organ‐specific metrics, mortality‐based metrics, and conventional aging metric. Accelerated organ aging leads to the diseases. Organ‐specific biological ages had significant positive correlations with each other. The organ specific biological age was associated with both organ‐specific and non‐organ specific diseases.
Tian et al. (2023)	Cross‐sectional (*n* = 143,423 for organs, *n* = 36,901 for brain) and longitudinal (organs: *n* = 1220, brain: *n* = 1294) UK Biobank	Trained chronological age on brain imaging of 3 regions, and organs' physiological measures and blood‐derived phenotypes	ICD‐10‐based 16 diseases, leukocyte telomere lengths, survival time, mortality	7 Body organ systems physiological measures and 3 brain imaging; difference between chronological age and predicted age, known as the age gap index across organs and brain. An organ's biological age selectively influences the aging of other organ systems as networks; they showed heterogeneity of biological aging across individuals and organs.
Salimi et al. (2025)	Longitudinal BLSA (*n* = 907), InCHIANTI (*n* = 986), and NHANES (*n* = 44,790)	Trained Body Organ Disease Number on severity of organ specific and all organs' diseases to develop organ specific clocks (BSC) and Body Clock, trained chronological age on the clocks to obtain rate of aging, Gold‐standard sub‐clinical, clinical, laboratory, echocardiography and electrophysiology examinations	93 health, sub‐clinical and clinically defined diseases, geriatric syndrome (urinary or bowel incontinence or injurious falls), disability items (47), SBPP, mortality	Intrinsic aging clocks (BSCs) and organ‐specific rates of aging (BSAs) across 13 organs demonstrated substantial heterogeneity both between organs and among individuals, with low inter‐organ correlations. The Body Clock represents a health entropy metric, while the Body Age reflects the overall rate of body aging. The Body Clock achieved over 90% accuracy in predicting disability, geriatric syndromes, and mortality. Subclinical organ‐specific conditions, such as early‐stage chronic kidney disease and subclinical hypothyroidism, contributed variably to health entropy. The Speed‐Body Clock captures the influence of the Body Clock on walking speed, and the Speed‐Body Age indicates its aging rate. Similarly, the Disability‐Body Clock reflects the Body Clock's association with physical and cognitive disability, while the Disability‐Body Age quantifies the corresponding rate of functional decline.

Abbreviations: BLSA, Baltimore Longitudinal Study of Aging; BSA, bodily system specific age; BSC, bodily system specific clock; ICD, International Classification of Diseases; InCHIANTI, Invecchiare in Chianti; MESA, multi‐ethnic study of atherosclerosis; NHANES, National Health and Nutrition Examination Survey; SBPP, short battery of physical performance.

They identified 856 organ‐enriched proteins from an initial set of 4778, which were used to train machine learning models that predicted chronological age based on organ‐specific protein signatures. To capture a broader aging signal, the researchers also developed an “organismal” aging model using the remaining 3907 proteins that were not specific to any one organ, aiming to reflect shared aging processes across tissues. In addition, for comparison, a “conventional” proteomic aging model was trained using all 4778 plasma proteins to evaluate how organ‐specific and global aging metrics aligned with previously reported systemic aging signatures (Lehallier et al. 2019; Tanaka et al. 2020, 2018). These findings provided compelling evidence that aging is a heterogeneous and organ‐specific process and that mapping circulating proteins to their tissue‐specific gene expression origins offers a powerful framework for estimating biological age (Table 1).

Goeminne et al. also used proteomic data from approximately 45,000 participants in the UK Biobank (UKBB) to train a proteomic clock based on 3000 plasma proteins measured by the Olink Explore 3072 platform (Goeminne et al. 2025). They developed proteomic‐based organ aging metrics to capture inter‐organ heterogeneity (Table 1). Following Oh et al., proteins were defined as organ‐specific if their expression in one organ, based on GTEx data, was at least fourfold higher than in all others. These organ‐specific proteins were then used to build models predicting chronological age, retaining only those with a consistent correlation with chronological age (*r* > 0.30) and mortality. They reported that the first‐generation brain‐, artery‐, and kidney‐specific models predicted chronological age slightly better than the models of Oh et al., while other organ models performed marginally worse. Although organ‐specific proteomics have provided valuable insights into disease mechanisms, the manifestation of disease phenotypes is not always driven by organ‐specific proteins and can depend on protein alterations in other organs. For example, reduced Klotho expression in the kidney has been linked to systemic disorders involving the heart (e.g., left ventricular hypertrophy (Chan et al. 2018; Edmonston et al. 2024)) and brain (e.g., Alzheimer's disease (Chen, Shao, et al. 2023; Wu, Lei, et al. 2023)). The organ‐specific biological age was associated with both organ‐specific and non‐organ‐specific diseases. However, excluding models with lower correlations to chronological age may risk overlooking younger individuals exhibiting accelerated biological aging.

Goeminne et al. modeled deviations from chronological age and found associations with single chronic diseases such as stroke, dementia, and heart failure. Additionally, they trained a mortality‐based proteomic model using Cox proportional hazards with elastic net regression and validated their findings in samples from the Multi‐Ethnic Study of Atherosclerosis (MESA). Importantly, they observed that mortality‐based biological age differed significantly between sexes, with men showing older proteomic age and higher mortality, an observation consistent with known sex disparities in mortality (Goeminne et al. 2025). This suggests that mortality‐derived biological age reflects not only the underlying chronic disease burden but also biological pathways potentially influenced by sex‐specific risk mechanisms (Crimmins et al. 2019). To disentangle such pathways, future studies could develop sex‐specific proteomic clocks trained on mortality and compare clock components across sexes.

Using physiological and blood‐derived phenotypes from the UKBB, Tian et al. developed biological age models for seven body organ systems (Table 1). They examined 16 chronic diseases and predicted mortality using organ‐specific biological aging markers across cardiovascular, pulmonary, musculoskeletal, immune, renal, hepatic, and metabolic systems (Y. E. Tian et al. 2023). They trained models to predict chronological age based on system‐specific phenotypes, generating sex‐specific biological ages in individuals aged 39–73 years. They defined “body age gap” as the difference between predicted age and chronological age. For reference, they used predicted chronological age in healthy individuals to establish population norms. An individual's organ system was then considered older (gap > 0) or younger (gap < 0) than the norm for their chronological age and sex. These age gaps served as normative, organ‐specific clocks of biological age. However, one critical aspect of defining “normative” was the selection of individuals without reported clinical disease. However, as Salimi et al. demonstrated, subclinical conditions that might be misclassified as normative, such as subclinical hypothyroidism and early‐stage kidney function decline, can significantly contribute to multi‐system morbidity.

Salimi et al. introduced a comprehensive framework for quantifying organ‐specific and systemic aging using intrinsic aging clocks and aging rate metrics, termed BSCs and BSAs, respectively. These metrics were developed using 93 health, subclinical, and clinically severe diseases to define Body Organ Disease Number (BODN), a measure that captures the number of organ systems affected by at least one subclinical or clinical deviation from health, with a range spanning from 1 to 13 systems (Table 1). Since the burden and progression of organ‐specific disease are unequal, the differential contribution of each organ system to overall BODN was modeled using Bayesian ordinal regression. This approach generated BSCs, serving as proxies for organ‐specific intrinsic aging clocks. Each BSC was used to estimate chronological age, providing the corresponding BSA. When all organ systems were included in the model, the resulting integrated metric was termed the Body Clock, representing a composite measure of health entropy, defined as random expansion of health decline. The Body Clock was used to estimate chronological age, yielding Body Age, a holistic indicator of systemic aging (Salimi et al. 2025).

**FIGURE 2:**
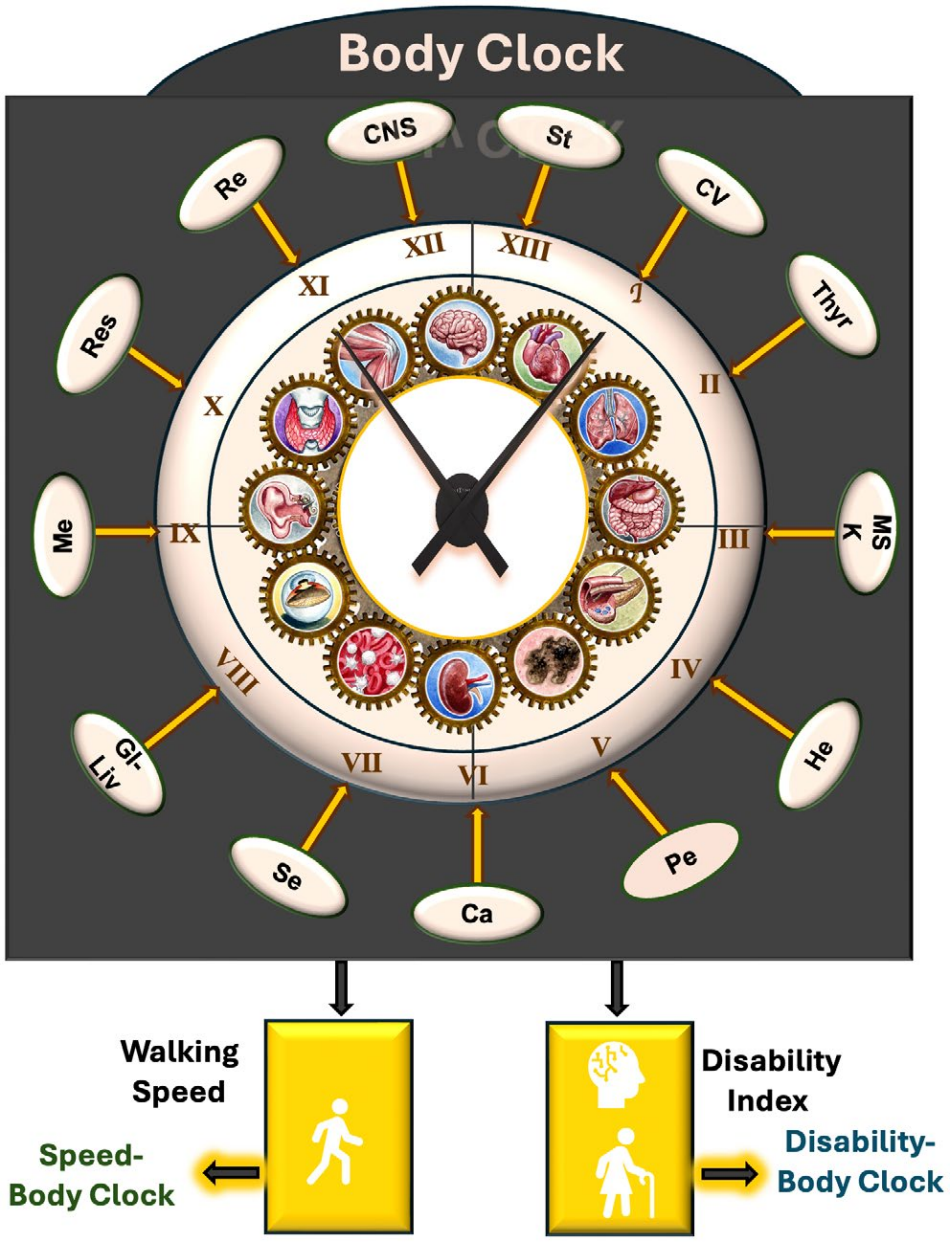
The Clock components of Health Octo Tool. (Salimi et al. 2025) Health Octo Tool include Bodily System Specific Clocks, Body Clock, Speed‐Body Clock, and Disability‐Body Clock. Each set of Clocks was regressed by chronological age to obtain estimated rate of aging for organs (Bodily System Specific Age and Body Age). CV, cardiovascular; Thyr, thyroid; MSK, musculoskeletal; He, hematopoiesis; Pe, periodontal health; Ca, cancer; Se, sensory; GI‐Liv, gastrointestinal and liver; Me, metabolic; Res, respiratory; Re, renal (kidney); CNS, central nervous system, St, stroke.

Furthermore, the Body Clock framework was extended to predict key functional outcomes, such as walking speed—a validated marker of aging phenotype—resulting in the Speed‐Body Clock. Disability Index was developed using 47 items of physical and cognitive disability. When the model was trained to predict the Disability Index, it yielded the Disability‐Body Clock. Each of these clocks was further translated into biological age metrics, regressing chronological age over the clocks, termed Speed‐Body Age and Disability‐Body Age, respectively (Table 1, Figure 2).

Together, these tools offer a novel, scalable, and interpretable approach to capturing the heterogeneity of biological aging. They lay the groundwork for future applications in personalized monitoring and early interventions targeting age‐related decline. In particular, by mapping aging trajectories at both organ‐specific and systemic levels, these metrics enable quantification of health entropy and aging acceleration.

Salimi et al. found that some organs, particularly the kidney and brain, often exhibit biological ages that can exceed chronological age, similar to findings from Oh et al., who reported that individuals can show accelerated aging in one or more organs (Oh et al. 2023; Salimi et al. 2025). These observations suggest that distinct biological pathways underlie aging in different organs and may be targets for interventions.

However, Oh et al.'s models did not include aging metrics for the thyroid or for bone‐related conditions such as osteoporosis and osteoarthritis, common drivers of late‐life morbidity and mobility (Oh et al. 2023). In contrast, Salimi et al. identified subclinical hypothyroidism as a key contributor to systemic aging, highlighting the importance of broader organ coverage even at early‐stage sub‐clinical changes in aging research. Of particular interest is the kidney, which both studies identified as a high‐impact organ in aging. Oh et al. showed that a larger kidney age gap (defined as the difference between biological and chronological kidney age) was significantly associated with hypertension and diabetes. However, Salimi et al. showed that kidney‐function decline with age can also occur independent of these risk factors and is often linked to chronic inflammation (“inflammaging”) (Salimi et al. 2018). This suggests that proteomic aging models trained on gene expression–mapped proteins may inadvertently reflect common multimorbidity disease burdens in the population (e.g., hypertension or diabetes). Salimi et al. provided further support for this interpretation by showing that age‐related subclinical kidney dysfunction (stage 1 CKD), independent of diabetes and hypertension, contributes significantly to system morbidity, as measured by the BODN. They identified the kidney as a sentinel organ for accelerated aging and emphasized that chronic kidney disease in the absence of diabetes or hypertension may reflect intrinsic biological decline linked to systemic inflammation (Salimi et al. 2018). They categorized diabetes co‐morbid with CKD as more severe levels of diabetes. Interestingly, stage 1 CKD had a larger contribution to BODN, emphasizing the initiation of the aging process at sub‐clinical stages (Salimi et al. 2025).

Additionally, Goeminne et al. also reported that individuals with advanced proteomic aging, “extreme agers,” had a greater burden of diagnosed chronic diseases, supporting the notion that mortality‐based clocks capture cumulative risk for age‐related morbidity (Goeminne et al. 2025). However, not all mortality is driven by multimorbidity; certain high‐risk conditions such as acute stroke or severe heart failure can contribute significantly to early mortality even in the absence of multiple comorbidities, underscoring the need to capture both disease burden and severity when modeling biological age (Salimi et al. 2025). These findings align with the broader concept of stochastic heterogeneity in aging. Chronic diseases vary not only in prevalence across individuals of the same chronological age but also in their severity and combination patterns across organ systems. This variability contributes to erratic responses to standard, single‐disease treatments and can result in polypharmacy and increased iatrogenic risk (Salimi et al. 2025).

While different studies considered organ systems aging assessments, the inclusions of organ systems have been rather specific in each study. For example, Tian et al. did not include thyroid status, sensory systems (e.g., vision and hearing), periodontal disease, heart failure, or ischemic heart disease and assigned metrics like body mass index, waist, and hip circumference to the musculoskeletal system while Salimi et al. considered the spectrum of normal to severe osteoarthritis, osteoporosis, and gout as part of the musculoskeletal system. Salimi et al.'s models incorporated 13 organ systems encompassing over 93 health states and calculated severity levels to assess contributions to the system morbidity. They also categorized diabetes and hyperlipidemia severity within the metabolic system (Salimi et al. 2025).

Tian et al. also found that advanced cognitive age correlated not only with brain aging but also with aging in other organ systems (Tian et al. 2023). Similarly, Salimi et al. showed that Body Clock could predict cognitive and physical disability. In particular, the Disability‐Body Clock and its cognate biological rate of aging, termed Disability‐Body Age, demonstrated that while some individuals in their 90s exhibited a Disability‐Body Age surpassing 130, others had a Disability‐Body Age as low as 60, manifesting resilience (Salimi et al. 2025).

**FIGURE 3:**
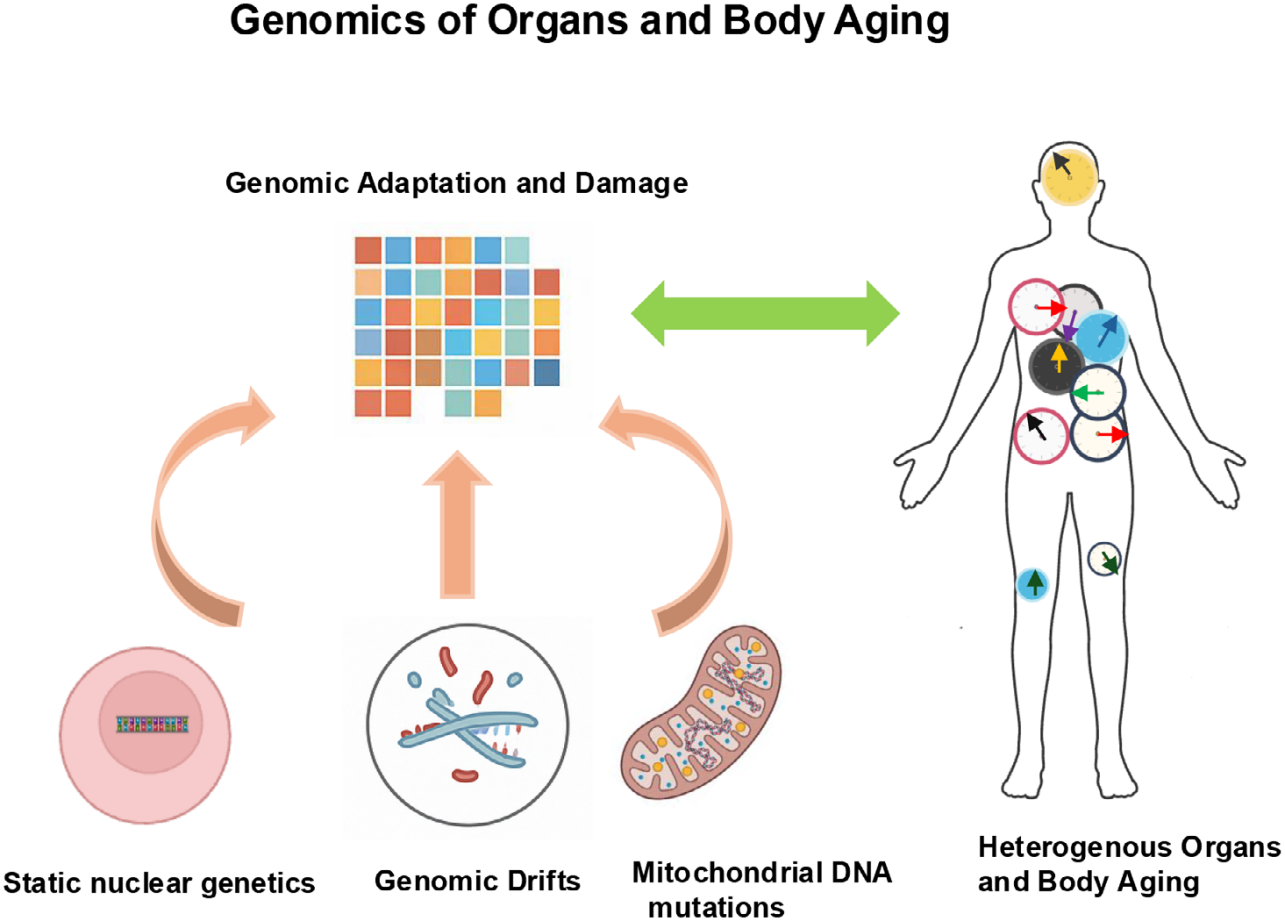
Static and dynamic genomic states, and heterogeneity of organ and body aging. Bodily system specific clocks, body clock and their rate of aging or proteomic‐based clocks can be used to determine static genetics and genomic drifts underlying these aging metrics.

Together, these findings reinforce the concept that aging, both at the organ and organismal level, is not uniform. We will discuss the possible static and dynamic genomic sources of organs and individuals' heterogeneity of aging (Figure 3).

## 2 Possible Genomic Contributions to the Heterogeneity of Organ and Body Aging

Genetic studies of chronic diseases have evolved substantially over the past century—from early family‐based linkage studies to modern genome‐wide association studies (GWAS) that have identified thousands of common variants associated with complex traits (Lander et al. 2001; Manolio et al. 2009). While GWAS has yielded important insights, it has also highlighted limitations: many common variants have small effect sizes, and a substantial portion of disease heritability remains unexplained (Boyle et al. 2017; Visscher et al. 2017). Increasingly, researchers have turned to other layers of genetic architecture, including somatic mutations, genomic instability, epigenetic changes, and mtDNA changes, such as mtDNA copy number and mutational burdens which have been linked to aging and age‐related diseases, such as cardiovascular disease, chronic kidney disease, and neurodegeneration (Chinnery and Hudson 2013; Malik and Czajka 2013; Tin et al. 2016; Yu 2012), with unknown causal roles (Bratic and Larsson 2013).

### 2.1 Static Genetic Variations

While aging itself is considered a stochastic process, germline genetic variation, here termed static genetic variation, might play a role in the heterogeneity in pace or time of aging in terms of proteomics or manifestations of organ‐specific complex diseases (Goeminne et al. 2025; Oh et al. 2023; Sacher 1982; Salimi et al. 2025; Tian et al. 2023). With the advent of whole‐genome sequencing, genetic studies of chronic diseases have begun to disentangle hereditary from sporadic genetic changes underlying single chronic diseases. While genetic studies have shown robust associations with single chronic diseases, such as *APOL1* in kidney disease (Friedman and Pollak 2020; Parsa et al. 2013) or the association of *FTO* and insulin resistance genes in obesity (Claussnitzer et al. 2015), among other examples (Guo et al. 2020), polygenetic risk scores explained single chronic diseases better than single nucleotides from genome‐wide association studies (Fahed and Natarajan 2023; Nurmohamed et al. 2024; Patel et al. 2023; Ravi et al. 2025; Small et al. 2024; Truong et al. 2024). However, the limited inclusion of admixed populations in both discovery and fine‐mapping individual‐level datasets poses a challenge for the development of polygenic risk scores and hinders their equitable clinical application across diverse populations (Ruan et al. 2025). With the advent of whole‐genome sequencing, the discovery of rare variants with larger effect sizes has become possible.

Notably, even in the presence of pathogenic mutations linked to specific diseases—such as BRCA1 associated with breast cancer—disease onset frequently occurs later in life. This pattern suggests that the penetrance and severity of certain genetic variants may increase with aging, potentially due to cumulative molecular damage, epigenetic drift, or age‐related environmental interactions. To explore this further, organ‐specific aging metrics can be applied to assess static genetic variants and investigate the mechanisms underlying their age‐dependent penetrance.

(Al‐Mulla et al. 2009). To elucidate the genetic basis of organ‐specific and body aging heterogeneity, organ aging metrics, and unitary metrics such as “conventional”, “organismal,” metrics (Goeminne et al. 2025; Oh et al. 2023), Body Clock, Body Age (Salimi et al. 2025) and intrinsic capacity, a composite measure of physical and mental capacities (Brodeau et al. 2025), can be utilized to investigate static genetic variants, thereby revealing the mechanisms that drive their age‐dependent penetrance.

Moreover, BRCA1 mutation occurs at high population frequency for three founder mutations in Ashkenazi Jews (King et al. 2003; Rosenthal et al. 2015); yet, the common deleterious static genetic variation cannot fully explain variable healthspan within expanded lifespan in this population. This is evidenced by the observation that the burden of pathogenic variants did not differ across groups. Specifically, disease‐associated mutation levels were comparable among exceptionally long‐lived individuals, their offspring, and control subjects without familial longevity, despite all originating from the Ashkenazi Jewish founder population (Gutman et al. 2020). This suggests stochastic and dynamic effects of environmental stressors and adaptive responses, yielding dynamic genomic alterations in the patterns of “switching on and off” of specific health‐related genes. Such dynamic alterations could be an accumulation of somatic mutations, transposon elements, mtDNA mutations, and dynamic signaling between somatic DNA mutation, DNA repair, and epigenetic drifts, among other changes (Ferrucci et al. 2020; Rosenthal et al. 2015; Vijg 2021).

Additionally, the study of rare variants suggests a negative impact for the accumulation of their mutations on healthspan and lifespan (Shindyapina et al. 2020). Rare genetic variants can persist in human populations when their introduction through mutation is balanced by natural selection. For example, in Alzheimer's disease (AD), numerous rare variants have been identified that are significantly associated with disease risk, including *TREM2*, *ABCA7*, and *SORL1*. These rare variants often have moderate to large effect sizes and contribute to the “missing heritability” observed in AD studies (De Deyn and Sleegers 2025; Lord et al. 2014). In general, the persistence of rare variants for disease can be attributed to several factors: (a) Late‐onset effects like AD that typically manifests after the age of 65. The deleterious effects of variants occurring post‐reproductively reduce the strength of natural selection against them. (b) *De novo* mutations: some rare variants arise as new mutations (dynamic genomics) in individuals, introducing novel genetic changes that can persist if not strongly selected against. (c) Incomplete penetrance and variable expression: not all individuals carrying a rare variant will develop pathology, and the severity can vary, allowing the variants to remain in the gene pool. As such, understanding the mechanistic role of rare variants in organ and body aging is crucial for unraveling a disease's complex genetic architecture and could inform the development of targeted interventions (Gibson 2012; Mani 2017). However, the associations of rare variants burden with heterogeneity of organs and body aging and whether their associations are causal and generalizable also require investigations. Development of polygenic risk scores of organ‐specific and body health metrics may provide insights into both shared and distinct genetic factors underlying heterogeneity in health outcomes and rates of aging.

## 3 Dynamic Genomic Alterations

### 3.1 Somatic Mutations

Somatic mutations and their accumulation over time result in tissue mosaicism and have been associated with age and environmental‐related chronic diseases (Huang et al. 2022; Maslov and Vijg 2023; Ren et al. 2024; Vijg and Dong 2020). There are few studies showing age‐related somatic mutation in human tissues associated with the heterogeneity of aging (Ren et al. 2022; Weinstock et al. 2023). Whether somatic mutations accumulation explain the heterogeneity of organ and body aging is unknown. Moreover, somatic mutation might not be entirely independent of static DNA sequences. There is evidence of interplay between static and stochastic genomes that suggests a possible role in the variability of disease manifestation within the same age group and between individuals. For example, clonal hematopoiesis of indeterminate potential (CHIP) has specific somatic mutations in the absence of other hematologic diseases, increases with aging and has been shown to be linked to atherosclerosis, inflammation, and cancer (Ebert and Libby 2018; Jaiswal and Ebert 2019; Jaiswal et al. 2017). CHIP has been associated with the mutated genes *DNMT3A* and *TET2*, which regulate epigenetic patterns (Buscarlet et al. 2017; Shlush et al. 2014). Somatic mutations in these genes have also been reported in 95% of healthy individuals aged 50–60 years, suggesting *de novo* somatic mutations in some genes with aging (Steensma 2018; Young et al. 2016). Whether these dynamic changes are adaptive or maladaptive with aging is not fully disentangled (Ren et al. 2024; Vijg et al. 2023).

### 3.2 Epigenetic Changes

One piece of evidence for an adaptation mechanism or disease development is provided by epigenetic changes. Methyl transferases (DNMT) transcription changes with aging and in cancers. Researchers have reported a decline in *DNMT1* expression levels accompanied by a steady increase in *DNMT3B* mRNA expression in aging cells, which is consistent with observed changes in protein production and enzymatic activity of these methyltransferases (Casillas Jr. et al. 2003). Alterations in the transcription of *DNMT1* and *DNMT3B* are likely key contributors to the global DNA hypomethylation and region‐specific hypermethylation observed in aging cells, while the expression of all *DNMTs* tends to be significantly upregulated in cancer cells (Casillas Jr. et al. 2003). Another study showed that the methylation enzyme Dnmt1 is employed to sites of oxidative DNA damage, an important aging mechanism (Jin and Robertson 2013). Recruitment of Dnmt1 leading to DNA methylation (DNAm) to inhibit transcription at the promoters of genes is a possible adaptation mechanism to DNA damage. Moreover, Dnmt1 is known to play a role in DNA stability, and its inactivation results in further DNA damage (Jin and Robertson 2013). In response to DNA damage, Dnmt1 is recruited to the site of recombinational repair, resulting in reversible or irreversible silencing in expression of the repaired genes (Ding et al. 2019). Such a phenomenon has been reported mainly in cancers (Jin and Robertson 2013). Thus, the study of the DNAm patterns in whole DNA genes that track with organ and body aging are warranted. Several epigenetic metrics, in particular Epigenetic Clocks have consistently captured age‐related changes (Belsky et al. 2020; Belsky et al. 2022; Hannum et al. 2013; Horvath 2015; Levine et al. 2018; Lu et al. 2022; Lu et al. 2019).

The first generation of epigenetic biomarkers of aging was developed by training CpG sites on chronological age as a surrogate for biological age (Hannum et al. 2013; Horvath 2015). One of the earliest examples is the Horvath Epigenetic Clock (Horvath 2013), which predicts not only time to death (Chen et al. 2016) but also the onset of specific chronic conditions such as renal function decline (Matias‐Garcia et al. 2021) and diabetes (Vetter et al. 2023).

A genetic variant in the telomerase reverse transcriptase gene (*TERT*), which is associated with longer leukocyte telomere length, has been reported to be independently associated with a DNAm aging clock (Lu et al. 2018), indicating an interplay between static genetic and epigenetic changes.

The second‐generation epigenetic clocks were trained jointly on chronological age and phenotypes. Among the second‐generation DNAm clocks, DNAm PhenoAge was trained on the PhenoAge measure—a composite score integrating physiological markers from multiple systems—and significantly outperforms first‐generation clocks in predicting all‐cause mortality, cancer incidence, morbidity burden, physical functioning, and Alzheimer's disease (Levine et al. 2018). DNAm GrimAge, trained on 88 plasma proteins including plasminogen activator inhibitor 1 (PAI‐1) and growth differentiation factor 15 (GDF15), and smoking‐pack‐year—associated with cardiovascular and other age‐related diseases—predicts diabetes, cardiovascular disease, renal function decline, and overall morbidity burden (Lu et al. 2019).

The updated DNAm GrimAge2 incorporated log‐transformed plasma protein markers for high‐sensitivity C‐reactive protein (log‐CRP) and hemoglobin A1C (log‐A1C). GrimAge2 demonstrated enhanced performance in predicting mortality compared to the original GrimAge and showed stronger associations with age‐related single traits such as coronary heart disease, reduced lung function, and fatty liver disease. Notably, DNAm log‐CRP correlated with morbidity, DNAm log‐A1C with type 2 diabetes, and DNAm PAI‐1 with triglyceride and visceral fat levels (Lu et al. 2022).

In parallel, Belsky et al. developed DunedinPACE, which trains DNAm at CpG sites on 19 indicators of organ‐system integrity to estimate *epigenetic aging*, effectively predicting lifespan and single diseases (Belsky et al. 2022).

Despite advances across the first and second generations of epigenetic clocks—many of which effectively predict specific single diseases or overall disease burden—none directly capture organ‐specific accelerated aging. Recently, the Levine group introduced the DNAm SystemAge, which trains CpG sites on physiological markers across 11 organ systems (Sehgal et al. 2025). However, many of these markers exhibit limited organ specificity, which complicates their interpretation in the context of organ‐specific aging. Moreover, certain systemic biomarkers (e.g., CRP, a marker of immune function) are influenced by static genetic variation, introducing potential effects of genetic heterogeneity (Raffield et al. 2020).

While some epigenetic clocks (e.g., the pan‐tissue Horvath clock) provide consistent age estimates across tissues, other epigenetic clocks (e.g., organ‐ or tissue‐specific clocks for brain, liver, or muscle) are required to reveal distinct aging trajectories reflective of organ‐specific biological variation. This distinction between organ‐specific and single unitary metrics underscores that epigenetic markers can capture both systemic and organ‐specific dimensions of aging, thereby improving our understanding of organ aging heterogeneity. Identifying epigenetic signatures linked to clinically defined organ‐aging phenotypes can further illuminate organ‐specific, age‐related epigenetic biomarkers and create hypotheses on mechanisms.

Moreover, the interplay of epigenetic changes and DNA variants known as methylation quantitative trait loci (meQTLs) decipher the interaction of static and dynamic genetics with DNAm. meQTL is a genomic locus where variation (typically a single nucleotide polymorphism [SNP]) is statistically associated with variation in DNAm levels at nearby (cis) or distant (trans) CpG sites. Mendelian randomization analyses showed association of cis‐meQTL variants with clonal hematopoiesis traits, particularly myeloid clonal hematopoiesis and the number of clonal mutations (Kirmani et al. 2025). Whether DNAm changes are repair or resilience mechanisms or play a role in disease development requires further scrutiny. One challenge is the differentiation of developmental‐related methylation patterns in CpG sites from *de novo* methylation changes. Such changes are in response to DNA damage through the stabilizing Dnmt1 protein or *de novo* Dnmt3a and Dnmt3b recruitment in response to extrinsic and intrinsic insults (Robert 2020). Such variability in the epigenetic landscape in response to DNA damage might result in heterogeneous phenotypes (Jin and Robertson 2013), but further study is required to elucidate the interplay between DNA sequencing variants, environmental exposome, DNA damage, and epigenetic changes tracking with organ and body metrics of aging (Dolcini et al. 2020; Sofer et al. 2013). One might even speculate on an adaptive role for the accumulation of mutations and epigenetic changes and their role in decreased compensation mechanisms to additional stressors, such as accumulation of exposomes (Baccarelli 2019).

### 3.3 DNA Damage and Repair

DNA double‐strand breaks (DSBs) are the most fatal type of DNA damage, and sirtuins and other chromatin modifiers have been shown to re‐localize to the site of the damage to prepare for repair and genome stability. Re‐localization of sirtuins (Tian et al. 2019), whose function is silencing repeat sequences and promoters, to the site of DNA damage can result in epigenomic drift and gene expression variability with aging (Oberdoerffer et al. 2008). Such adaptation machinery for DNA damage amelioration might be exhausted at a variable rate over lifespan when coupled with random epigenetic drift over time (Bahar et al. 2006). Whether DNA scars, together with increased methylation at sites of DNA damage, contribute to variability in health outcomes and disease—and consequently to organ‐ and individual‐level aging heterogeneity—remains to be determined. Addressing this question will require integrated epidemiological studies with available datasets, complemented by experimental organ‐ and tissue‐specific investigations. For example, Brodeau et al. showed association of DNA damage markers, the number of γ‐H2AX foci per nucleus, and chronological age and health (Brodeau et al. 2025). Researchers reported that the successful repair response to nonrandom DSBs was accompanied by an accelerated Horvath Epigenic Clock and resulted from re‐localization of sirtuins in an inducible change in an epigenome (ICE) mouse model (Yang et al. 2023). This suggests that some epigenetic patterns can be due to an adaptation response to DNA damage. With aging, Sirt1 levels decline in response to increased reactive oxygen species (ROS) accumulation (Hwang et al. 2013), which impairs heterochromatin‐mediated gene silencing, contributing to age‐related changes in gene expression. Moreover, ROS influences DNA demethylation both through oxidative damage to DNA and via modulation of ten‐eleven translocation (TET) enzyme–mediated hydroxymethylation (Chia et al. 2011). Although alterations in TET enzymes and age‐related epigenetic drift have been linked to individual chronic conditions (Borkowska et al. 2020; Dzitoyeva et al. 2012; Ferrone et al. 2020; Hadad et al. 2016; Huang et al. 2020; Kolodziej‐Wojnar et al. 2023; Solary et al. 2014; van den Hove et al. 2012; Zhang et al. 2024), their contribution to organ‐specific heterogeneity in aging has not yet been investigated. As such, the hypothesis remained to be addressed whether variability in CpG density as an epigenetic erosion, resulting from chromatic modifier recruitments at the site of DNA damage, explains variability in rates of organ and individual aging (Bertucci and Parrott 2020).

Additionally, ROS that induces DNA damage can indirectly impact epigenetic regulation by altering the availability of key metabolites, such as acetyl‐CoA, iron, α‐ketoglutarate, NAD^+^, and S‐adenosylmethionine, which are critical metabolites for histone‐modifying enzymes. These observations highlight the close interplay between epigenetic modifications and the cell's metabolic and energetic state (Simpson et al. 2012). Of note, there is a hypothesis that the accumulation of such molecular alterations may serve an adaptive role, representing a decline in compensatory capacity to environmental and endogenous stressors, including the accumulation of multiple environmental exposures (exposome) (Juarez et al. 2020; Wu, Eckhardt, and Baccarelli 2023).

Moreover, there is evidence for an association between DNA repair impairment and single chronic diseases, based primarily on models of accelerated aging such as progeria and Werner syndromes (Oshima et al. 2017). While DNA repair damage has been associated with accelerated aging in human and animal models, another DNA‐based theory could also be involved. The *two‐hit hypothesis* suggests that both alleles, for example, tumor suppressor genes such as Rb, can be inactivated either through mutations or through epigenetic silencing to cause a phenotypic change (Park et al. 2021). Although this mechanism has been shown for cancers such as retinoblastoma, whether the underlying hypothesis through *de novo* somatic mutation or epigenetic silencing plays a role in developing age‐related diseases and/or organ and body aging, as well as in the context of germline genetic structure, needs to be investigated. Alternatively, the effect of some genes might be dose‐dependent so that epigenetic or genetic changes in one copy might result in disease phenotypes, a mechanism known as haploinsufficiency (Abdelfattah et al. 2021; Brown et al. 2007; Cabelof et al. 2006; Celaya et al. 2021; Goldman et al. 2005; Hathcock et al. 2002; Kurzhals et al. 2011; Mehalko et al. 2023; Mendias et al. 2015; Moreno‐Cugnon et al. 2018; Park et al. 2016; Patterson and Cabelof 2012; Schmutz et al. 2020; Sedic et al. 2015; Thakur et al. 2017). Notably, in addition to mutations in genes that directly regulate DNA repair, mutations in genes indirectly involved in DNA repair can also impact response to DNA damage. For instance, recent research has shown that fat mass and obesity‐associated protein (FTO) may weaken DNA repair by reducing the activity of poly (ADP‐ribose) polymerase 1 (PARP1). Recruitment of PARP1 protein to DNA damage sites represents one of the earliest processes in multiple DNA repair pathways in response to DNA damage (Zhu et al. 2025). Moreover, enhanced activation of DNA repair mechanisms may contribute to biological resilience. Also, in a study conducted by Jan Vijg's group, smoking was associated with an increased burden of somatic mutations in human bronchial epithelial cells. Notably, among very heavy smokers—those who seemingly survived the deleterious effects of long‐term smoking—a paradoxically lower rate of somatic mutations was observed, suggesting the presence of compensatory mechanisms such as heightened DNA repair activity or potential underlying protective static genetic variants (Huang et al. 2022). Such mechanisms may also underlie heterogeneity of aging trajectories. For instance, some individuals in their 90s may have a Disability‐Body Age of 65, indicating slower aging, while others in their 80s may exhibit a Disability‐Body Age of 130, reflecting accelerated aging (Salimi et al. 2025).

### 3.4 Retrotransposable Elements (RTEs)

RTEs are also part of stochastic genomics. They are repetitive DNA sequences widely distributed across eukaryotic genomes. With age, the silencing mechanisms that normally suppress RTEs tend to weaken, leading to their aberrant activation. This reactivation has been observed in multiple studies and is linked to reduced healthspan (Li et al. 2013; Macciardi et al. 2022). Among the RTEs, LINE elements are particularly active in mammals. Notably, species that fail to tightly control LINE activity exhibit shorter lifespans than those that maintain stringent regulation (Lander et al. 2001).

RTE reactivation is implicated in the onset of various aging phenotypes and age‐related diseases. It is associated with increased cellular senescence, inflammation (De Cecco et al. 2019), heterochromatin disorganization (Della Valle et al. 2022), and genomic instability driven by epigenetic changes (Erwin et al. 2016).

Under normal conditions, RTEs are epigenetically silenced through mechanisms such as DNAm (Li et al. 1993), histone methylation (Nakayama et al. 2001), and histone deacetylation mediated by enzymes like Sirt6 and Sirt7 (Kawai and Montgomery 1987; Van Meter et al. 2014). These epigenetic defenses begin early in development through *DNMT3* and are maintained in somatic cells by *DNMT1* (Deniz et al. 2019; Karimi et al. 2011; Walsh et al. 1998). However, these pathways deteriorate with age, leading to loss of epigenetic regulation and RTE reactivation.

Aging also impairs chromatin structure and transcriptional silencing, particularly in intergenic regions rich in RTEs. These changes are marked by a loss of H3K9me3, a histone mark associated with stable heterochromatin, in aged cells (Pal and Tyler 2016; Sen et al. 2016).

Furthermore, RTE reactivation and heterochromatin loss are observed not only in aged cells but also in younger individuals with age‐related phenotypes (De Cecco et al. 2013; Fraga et al. 2005; Villeponteau 1997) and in accelerated aging syndromes such as Werner and progeria (Della Valle et al. 2022; Zhang et al. 2015). Evidence from monozygotic twin studies shows that age‐related loss of DNAm at RTE CpG islands is more pronounced in the twin with a less healthy lifestyle (Fraga et al. 2005; Martin 2005; Talens et al. 2012). The RTEs' associations with organ and body aging heterogeneity and organ‐specific causal effects require investigations.

### 3.5 Mitochondrial DNA

MtDNA, which is maternally inherited, lacks introns and exists in high copy numbers in cells with high metabolic demands (Stewart and Chinnery 2015; Wallace 2015).

Mitochondria are essential for maintaining energy production, cellular homeostasis, and redox balance (Jang and Van Remmen 2009; Li et al. 2025). As organisms age, mitochondrial efficiency declines, leading to reduced mitochondrial coupling, reduced ATP synthesis (Marcinek et al. 2005), and increased production of ROS. Excess production of ROS, as byproducts of impaired oxidative metabolism, can cause oxidative damage to critical cellular components, including mtDNA, proteins, and lipids. mtDNA is especially susceptible due to close exposure to ROS and its higher mutation rate, lack of histone proteins and limited repair capacity compared to nuclear DNA (Dirks et al. 2006; Srivastava 2017). Although mtDNA mutations have been correlated with numerous age‐related diseases (Bonawitz and Shadel 2007; Dirks et al. 2006), experimental studies are required to assess whether the gradual accumulation of mtDNA mutations contributes to tissue‐specific phenotypes malfunction and heterogeneity of organ aging. The effects of mtDNA changes on mitochondrial dysfunction such as impaired oxidative phosphorylation, increased oxidative stress (Dai et al. 2014; Schriner et al. 2005), reduced activity of metabolic enzymes, changes in metabolite levels (Srivastava 2016, 2017), altered mitochondrial overall morphology, dynamics, and biogenesis (Dai et al. 2017; Kauppila et al. 2017; Sun et al. 2016) require further organ‐specific experiments. Moreover, mitochondria undergo regular cycles of fission and fusion during their functioning, so each cell has its own mitochondrial morphology at any given time. Whether the number and shape of mitochondria are related to changes in mtDNA mutations across organs as well as mitochondrial mosaicism within the same tissue are unknown (Chen, Zhao, and Li 2023; Chen and Zweier 2014; Sarver et al. 2024; Soong et al. 1992; Srivastava 2017). The aging process is thought to be influenced in part by this cycle of mitochondrial damage although the causal relationships are still speculative (Bonawitz and Shadel 2007; Meissner 2007; Poulose and Raju 2014). Therefore, investigating tissue‐specific mitochondrial phenotypic and functional changes in response to mtDNA mutations or variations in copy number is essential to determine whether these mechanisms contribute to organ and body aging heterogeneity.

In the mitochondria, somatic mutations accumulate with age. Thus, mtDNA has been investigated as a biological clock to predict aging (Harman 1972), and its copy number, mutations and dysfunction have been associated with age‐related phenotypes (Bazzani et al. 2022; Bose and Beal 2016; Desdin‐Mico et al. 2020; Ferrucci et al. 2020; Filograna et al. 2021). The rate of mtDNA mutations and DNA copy number can be used to assess the rate of heterogeneity of organs and body aging. However, whether mtDNA copy number is an adaptive mechanism or deleterious (Filograna et al. 2021) needs to be examined in longitudinal and interventional studies targeting these mechanisms. Moreover, the potential causal relationship between mtDNA copy number and functional alterations in tissues and organs warrants investigations.

## 4 Summary

Heterogeneity in the aging process is key to understanding the mechanisms of variability of organ and body aging. Recent research has shown the heterogeneity of body organ systems combinations that suggests multifaceted mechanisms with possible variability of both static and stochastic states at all genomic levels in the same organ and/or across organs.

Metrics of intrinsic aging and rate of aging, such as ageotypes, BSCs, BSAs, Body Clock and Body Age and other components of Health Octo Tool such as Speed‐Body Clock and Disability‐Body Clock and their rate of aging, can be used to track such biomarkers (Goeminne et al. 2025; Oh et al. 2023; Salimi et al. 2025).

The extent to which stochastic genome and epigenome states at the DNA, mtDNA, and RNA levels are reversible by interventions such as calorie restriction, exercise, or anti‐aging drugs will shed light on possible mechanisms. For example, in patients with late chronic kidney disease, exercise did not improve mitochondrial function (Watson et al. 2020), suggesting a possible benefit of early intervention in preventing CKD at earlier stages. As Salimi et al. showed, early‐stage CKD at sub‐clinical level significantly contributed to BODN, a metric of system morbidity. Moreover, a study employing three of the four Yamanaka factors, including Oct3/4, Sox2, and Klf4 at transcription start sites, showed epigenetic reprogramming reversing the aging process in retinal cells in a mouse model (Y. Lu et al. 2020; Takahashi and Yamanaka 2006). This transcription factors bind to transcription start sites of the genes whose promoters are hypomethylated. While epigenetic reprogramming has been suggested as a possible reversal mechanism and some epigenetic patterns are amenable to change, whether hypermethylation in response to DNA damage and the genes with silenced motifs for these transcription factors can be reversed needs to be examined. Moreover, whether reprogramming can rescue declining of other organs' function with aging is uncertain. Other enigmas also remain. Can reversible epigenetic programming alter other mechanisms of aging, such as accumulated somatic mutation and DNA repair impairment? Can individuals regain normal function after severe organ morbidities? Possible solutions can be assessed with experimental and epidemiological examinations of organ systems and whole body clocks and other tools for assessing organ and body aging rates in response to interventions (Ryan et al. 2025; Waziry et al. 2023).

While multiple omic‐based clocks have been introduced to predict single diseases and mortality (Belsky et al. 2022; Horvath 2015; Levine et al. 2018; Lu et al. 2022; Lu et al. 2019), the Health Octo Tool as a clinical‐based tool was unveiled as a comprehensive multi‐dimensional health metric, encompassing organ‐specific and body intrinsic clocks and their rate of aging, which in turn can be used as a base to develop allocated multi‐omics clocks and identify organ‐specific and whole body pathways for each individual (5). These metrics can be used to reveal sources of variability in underlying mechanisms, determining a personalized intrinsic rate of aging at different ages and personalized responses to the impact of intervention. Addressing heterogeneity in the aging of organs and individuals is a key to developing precision clinical tools.

## Author Contributions

Shabnam Salimi lead the project, conducted the comprehensive literature review, and developed the conceptual framework and content for each section of the manuscript. Luigi Ferrucci provided substantial input on the biological mechanisms of aging, contributed to the interpretation of findings, and was deeply involved in revising and refining the manuscript. Daniel Raftery offered critical feedback on the texts and figures, mechanisms of aging, was involved in revising the manuscript, and contributed to the editing and final review of the manuscript.

## Funding

This work was supported by the National Institute on Aging, k01ag059898 and P30AG013280.

## Conflicts of Interest

Shabnam Salimi has patent‐pending on algorithms of Health Octo Tool. The other authors declare no conflicts of interest.

## Data Availability

The authors have nothing to report.
